# The Effect of Proprioceptive Training on Technical Soccer Skills in Youth Professional Soccer

**DOI:** 10.3390/medicina61020252

**Published:** 2025-02-01

**Authors:** Meriç Eraslan, Alper Cenk Gürkan, Serhat Aydın, Musa Şahin, Seyfullah Çelik, Mehmet Söyler, Tolga Altuğ, Mustafa Alper Mülhim

**Affiliations:** 1 Faculty of Sport Sciences, Akdeniz University, Antalya 07058, Türkiye; mericeraslan@akdeniz.edu.tr; 2 Vocational School of Healthy Services, Gazi University, Ankara 06510, Türkiye; 3 Department of Physical Education and Sports, PhD Program, Institute of Health Sciences, Gazi University, Ankara 06570, Türkiye; serhataydingazi@gmail.com; 4 Faculty of Sport Sciences, Karabük University, Karabük 78050, Türkiye; musasahin@karabuk.edu.tr; 5 Faculty of Sport Sciencesi, Yıldırım Beyazıt University, Ankara 06800, Türkiye; seyfullahcelik@aybu.edu.tr; 6 Vocational School of Social Sciences, Çankırı Karatekin University, Çankırı 18200, Türkiye; mehmetsoyler@karatekin.edu.tr; 7 Faculty of Sports Sciences, Ağrı İbrahim Çeçen University, Ağrı 04100, Türkiye; taltug@agri.edu.tr; 8 Faculty of Sports Sciences, Bolu İzzet Baysal University, Bolu 14280, Türkiye; mustafaalper.mulhim@ibu.edu.tr

**Keywords:** regular physical activity, soccer performance, proprioceptive training, exercise physiology, rehabilitation techniques, neuromuscular control, athletic development

## Abstract

*Background and Objectives:* This study analyzes the effects of proprioceptive training on the physical fitness and soccer-specific technical skills of young professional soccer players. *Materials and Methods:* Twenty-eight male professional soccer players from the Türkiye Football Federation’s Third League, aged 20.46 ± 1.60 years (average), participated voluntarily. Body composition was assessed with the Inbody270 bioelectrical impedance analyzer, while agility and maximal VO_2_ cardiorespiratory fitness were measured using the ChronoJump smartspeed mat. Soccer-specific technical skill assessments were also conducted. Data were analyzed using SPSS 22, with paired sample *t*-tests for comparisons, Cohen’s d test for effect size, and repeated measures two-way ANOVA for interaction effects (group × time). Eta squared (η^2^) values were reported for effect size. A 95% confidence level and *p* = 0.05 were used. *Results:* Proprioceptive training significantly improved body fat percentage (*p* < 0.05; η^2^ = 0.006) and soccer-specific technical skills, including free juggling (*p* < 0.05; η^2^ = 0.302), alternating foot juggling (*p* < 0.05; η^2^ = 0.271), right foot juggling (*p* < 0.05; η^2^ = 0.250), and shooting performance (*p* < 0.05; η^2^ = 0.513). *Conclusions:* A 12-week proprioceptive training program, when incorporated into soccer practice, significantly enhances soccer-specific technical skills and reduces body fat percentage.

## 1. Introduction

Soccer, as the most widely played and watched sport in the world, demands players maintain high levels of physical, technical, and mental performance to cope with the ever-increasing intensity and complexity of modern football. The structure of a match, consisting of two 45 min halves, requires players to sustain prolonged physical effort, with only a 15 min break in between. During this period, players must endure continuous exertion that demands both aerobic and anaerobic capacities for sustained performance [1]. The evolution of modern soccer tactics has increased the intensity, frequency, and quality of movements, placing a greater demand on players to perform at higher intensities [2]. As soccer continues to develop, it becomes essential for athletes to repeatedly cover short distances at high speeds, while also being able to perform technical skills under pressure.

While the specific movements of players are determined by their position on the field, soccer generally requires players to engage in high-intensity actions such as sprinting, dribbling, changing direction, and pressing. Explosive movements such as sprinting and quick changes in direction are essential across all positions, and this requirement persists regardless of the role of the player [3]. The game’s demands have evolved in such a way that soccer players now regularly experience bursts of high-intensity effort, followed by short periods of lower intensity, which requires them to have both endurance and explosive power [4].

The physical demands of soccer are compounded by the necessity for players to execute technical skills with high precision under conditions of fatigue [5]. As players become fatigued throughout the game, maintaining technical proficiency such as passing, shooting, and dribbling becomes increasingly challenging. Consequently, it is vital for soccer players to improve their physical conditioning and technical skills, which are crucial for performance and injury prevention [6]. Training for both physical conditioning and technical performance should be comprehensive, with a focus on strength, agility, balance, and coordination [7]. In addition to enhancing physical performance, improving proprioception—the body’s ability to sense its position in space—is essential for achieving optimal technical execution and decision-making on the field.

Proprioceptive training plays a crucial role in enhancing neuromuscular control, balance, and coordination, all of which are necessary for effective and efficient movement during soccer matches. Athletes with well-developed proprioceptive abilities are better equipped to respond to rapid changes in movement and environment [8]. Research has shown that proprioceptive exercises improve athletic performance by helping players maintain stability, adjust posture quickly, and execute technical skills under pressure [9]. Given the increasing demands for agility, reaction time, and decision-making under stress in modern soccer, proprioceptive training is becoming more widely recognized for its potential to improve technical performance and prevent injury [10].

Although proprioceptive exercises have been studied in various sports, there is limited research specifically exploring the effects of proprioceptive training on young soccer players’ physical fitness and technical performance [2]. Studies on proprioceptive training have mainly focused on its benefits for injury prevention and rehabilitation [6], with fewer investigations into how it affects specific technical skills like dribbling, passing, and shooting. This gap in the literature highlights the need for further research into how proprioceptive training can be applied to enhance the soccer-specific abilities of young athletes [11].

This study aims to investigate the effects of proprioceptive training on the physical fitness and soccer-specific technical skills of young soccer players. The primary hypothesis is that proprioceptive training will lead to significant improvements in both physical and physiological performance, ultimately enhancing technical skills and overall match performance. By addressing this gap in the literature, this research seeks to provide valuable insights into the role of proprioception in optimizing the performance of young soccer players.

## 2. Materials and Methods

### 2.1. Sample Characteristics, Calculation and Study Participants

This study involved 28 young professional soccer players from the Türkiye Professional Third League. The participants were selected based on specific inclusion criteria, with each player having a minimum of five years of formal training experience in soccer. The research is confined to the data collected from these 28 individuals during two distinct periods: the pre-season and the competitive season. Prior to participation, all players underwent thorough screening, with none reporting any history of cardiopulmonary conditions or the use of any medication during the study. Additionally, all participants had successfully passed the mandatory medical examination required for professional soccer involvement, ensuring their physical fitness for the demands of the study. The study protocol, including its potential risks and benefits, was clearly communicated to all relevant parties, including the participating clubs, coaches, and players. In accordance with ethical guidelines, all players provided written informed consent before taking part in the study, acknowledging their understanding of the study’s procedures and risks. This approach ensured that all ethical considerations were thoroughly addressed, safeguarding the participants’ rights and well-being throughout the research process.

Based on the findings of previous studies [12], we conservatively assumed a medium effect size of 0.75, which is commonly considered a practical and reliable estimate in the context of studies examining similar interventions. This effect size was chosen to reflect a moderate but meaningful impact that would be detectable within the scope of the study design.

To ensure statistical rigor, an alpha level of 0.05 (2-tailed) was selected, which is a standard threshold in hypothesis testing, indicating a 5% risk of committing a Type I error (i.e., rejecting the null hypothesis when it is actually true). Additionally, to minimize the risk of Type II error (failing to detect a true effect), a power of 0.80 was established, which is typically considered adequate for achieving a reasonable likelihood of detecting the effect if it truly exists. A power of 0.80 corresponds to an 80% probability of correctly rejecting the null hypothesis when it is false.

Given these statistical parameters, the sample size was calculated to ensure that the study would have sufficient power to detect meaningful differences between the groups. Specifically, based on the chosen effect size, alpha level, and power, it was determined that a minimum of 20 participants per group would be necessary to achieve reliable results. This sample size calculation helps ensure that the study’s conclusions are robust and that the findings can be generalized with confidence to similar populations. The required sample size was rounded to account for potential dropouts or incomplete data, with the final recruitment goal set higher to maintain statistical power.

The study included 28 professional male soccer players from the Türkiye Football Federation’s 3rd division, with an average age of 20.46 ± 1.60 years, an average height of 182.89 ± 5.01 cm, and an average body weight of 73.34 ± 3.10 kg. These players were randomly assigned to one of two groups: a control group consisting of 14 players (age: 20.81 ± 1.66 years; height: 183.29 ± 4.38 cm; weight: 73.08 ± 3.85 kg) and an experimental group also comprising 14 players (age: 20.07 ± 1.49 years; height: 182.50 ± 5.72 cm; weight: 73.60 ± 2.24 kg). The two groups were matched for baseline characteristics, with the only distinguishing factor being the additional proprioceptive training program incorporated into the experimental group’s routine. Aside from their standard soccer-specific training, the experimental group participated in a proprioceptive training program designed to enhance neuromuscular control, while the control group followed only the usual training regimen. This design allowed for the assessment of the effects of proprioceptive training on physical performance and technical skills, with both groups undergoing similar soccer-related activities.

Prior to the commencement of the study, participants completed the “Athlete Information and Measurement Form”, which documented their descriptive characteristics and confirmed that they were free from medical conditions, not currently using any medications, and had not participated in any proprioception or balance training programs in the last six months. All participants were fully briefed on the study’s objectives and methods in verbal sessions. Furthermore, to ensure informed participation, all athletes provided written consent by signing an “Informed Consent Form”, which outlined the purpose of the study, the procedures involved, and the potential risks, ensuring compliance with ethical research standards (Table 1).

### 2.2. Reliability and Validity of Tests

To ensure the robustness of the study and the reliability of the results, a comprehensive approach was used in selecting and validating the tests for assessing physical performance and technical skills. This section outlines the reliability and validity information for each of the tests conducted as part of the study.

#### 2.2.1. Physical Performance Tests

Yo-Yo Intermittent Recovery Test (Level 1): The Yo-Yo Intermittent Recovery Test was used to assess players’ aerobic capacity, simulating the intermittent running demands typical of soccer. This test has demonstrated excellent reliability and validity in previous studies with soccer players [13], and is widely regarded as one of the most reliable indicators of aerobic fitness in team sports. The test was conducted in accordance with standardized protocols, where participants completed repeated 20 m shuttle runs at increasing speeds, interspersed with short recovery periods. Reliability: The Yo-Yo Intermittent Recovery Test has been shown to exhibit high test-retest reliability, with intra-class correlation (ICC) values typically ranging from 0.85 to 0.92 in soccer populations [14]. Validity: The test’s validity has been established through strong correlations with maximal oxygen uptake (VO_2_ max) in soccer players [13], validating its use as an indicator of aerobic fitness.

30-Meter Sprint Test: The 30 m sprint test was employed to measure explosive speed, a key component of soccer performance. This test has demonstrated excellent test–retest reliability and has been widely used in soccer performance assessments. Reliability: Previous studies have reported excellent reliability for the 30 m sprint, with ICC values above 0.90 in soccer players [15]. Validity: The 30 m sprint test has been validated through its strong correlations with match performance outcomes such as sprinting distance and acceleration [16].

#### 2.2.2. Technical Skills Tests

Dribbling, Passing, and Shooting Tests: Specific soccer drills designed to assess dribbling, passing accuracy, and shooting were used to measure technical proficiency. These tests were chosen based on their common use in soccer training and performance assessments. Reliability: The reliability of these technical skill assessments has been supported by studies showing consistent results across repeated measurements in professional players [17]. For instance, the passing test demonstrated excellent intra-rater reliability (ICC = 0.88) and inter-rater reliability (ICC = 0.83) [18]. Validity: The validity of these skill assessments is established through their strong correlation with match performance and coaches’ ratings of player skill [19]. For example, shooting accuracy has been shown to predict success in game situations where players must perform under time pressure [20].

### 2.3. Statistical Parameters

In line with the chosen sample size, the reliability of the study was further ensured through rigorous statistical analysis. The study employed a medium effect size of 0.75, based on findings from similar research [21,22], ensuring that the power of the study (0.80) was sufficient to detect meaningful differences in physical and technical outcomes between the experimental and control groups. Reliability of the Sample Size Estimate: The power analysis conducted before the study ensured that the sample size was adequate to achieve reliable and valid results.

Given the parameters of effect size, alpha level (0.05), and power (0.80), the calculated sample size of 28 players (14 in each group) was deemed statistically sufficient to maintain confidence in the study’s outcomes.

### 2.4. Experimental Design

Participants were instructed to refrain from strenuous activities and ensure 24 h of rest prior to each testing session to avoid potential fatigue or interference with performance. They were also advised to maintain consistent dietary habits throughout the study to eliminate any dietary variations that could affect the outcomes. Specifically, participants were instructed not to alter their daily eating routines or use dietary supplements during the study period. They were asked to avoid fast food, alcoholic beverages, and foods high in sugar, while ensuring their diet remained balanced with meals containing vegetables, protein, and complex carbohydrates. Additionally, participants were required to consume their meals at the same times and in similar quantities. To further ensure uniformity across all testing sessions, all participants wore identical soccer boots equipped with rubber studs, minimizing footwear variability and ensuring a fair comparison of performance. The testing protocol followed a randomized order of assessments, which included body composition analysis, Illinois agility test, Yo-Yo Intermittent Recovery Test Level 1 (Yo-Yo IR1), maximum VO₂, and technical soccer skills. This randomization was intended to minimize the impact of fatigue or practice effects on performance across the different tests (Table 2).

Warm-Up Protocol: On each testing day, participants began with a standardized warm-up procedure designed to prepare their bodies for the physical demands of the assessments. The warm-up consisted of 5 min of jogging, followed by 5 min of joint mobility exercises aimed at improving flexibility and range of motion. This was followed by three progressively faster 30 m sprints to prime the muscles for high-intensity efforts [23]. Testing sessions were conducted on a natural grass soccer field, with evaluations taking place both in the morning and afternoon. The ambient temperature during testing was carefully controlled, ranging between 20 and 22 °C, ensuring optimal conditions for performance. Participants wore form-fitting athletic attire, which allowed for ease of movement, and their own indoor soccer shoes were used to standardize footwear across all tests.

All evaluations were conducted at the facilities of the participating soccer clubs, which provided access to on-field settings for assessments related to body composition, Illinois agility, Yo-Yo IR1, maximum VO_2_, and technical soccer skills. Each assessment was carried out with two players per group, ensuring that the equipment was consistently placed in the same location for each trial, thereby minimizing external variables.

**Figure 1:**
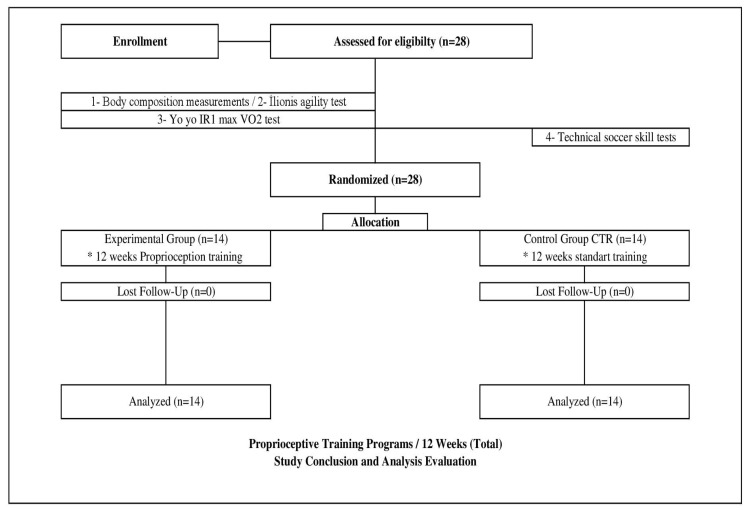
Research Design Flowchart. *: indicates: 12 weeks proprioception training and 12 weeks standard training.

Data collection was organized systematically in pairs, with at least two trained test administrators present at all times to oversee the assessments, optimize efficiency, and maintain the integrity of the testing procedures. This approach ensured that all participants were tested under controlled and consistent conditions, promoting reliable and valid data collection throughout the study (Figure 1).

### 2.5. Testing Procedures

#### 2.5.1. Body Composition Examination

Participants reported to the laboratory for height measurement, which was conducted using a stadiometer (Holtain Ltd., Crymych, UK) with precision to the nearest 0.001 m. Body composition, including body weight (BW) and body fat percentage (BFP), was assessed using the In-Body 270 device (Hologic QDR Series, Delphi A model, Bedford, MA, USA) with Hologic APEX software (Version 13.3:3, Hologic, Bedford, MA, USA), in line with the manufacturer’s recommended procedures. Participants were instructed to arrive in a rested, fasted, and hydrated state, and to refrain from engaging in strenuous physical activities for 24 h prior to testing to minimize any potential impacts on the results [24].

To standardize the testing conditions, participants wore only shorts and were required to remove any metal objects and jewelry before the assessment. Prior to each measurement session, the InBody 270 device was calibrated daily using phantoms, in accordance with the manufacturer’s guidelines, to ensure accuracy and consistency of results. Participants assumed a stationary, supine position on the scanning table, with their hands placed level with their hips and feet slightly apart, as described in a recent study [25].

All scanning and analysis procedures were performed by the same trained operator to maintain reliability and minimize inter-rater variability, following standardized testing protocols recognized as best practice. Whole-body data were recorded, excluding the head, and the measurements were consistently taken at 10:00 AM to control for any potential diurnal variation in body composition [26]. These meticulous protocols were implemented to ensure the reliability and accuracy of the body composition assessments across all participants.

#### 2.5.2. Illinois Agility Test (with Ball)—İA

**Figure 2:**
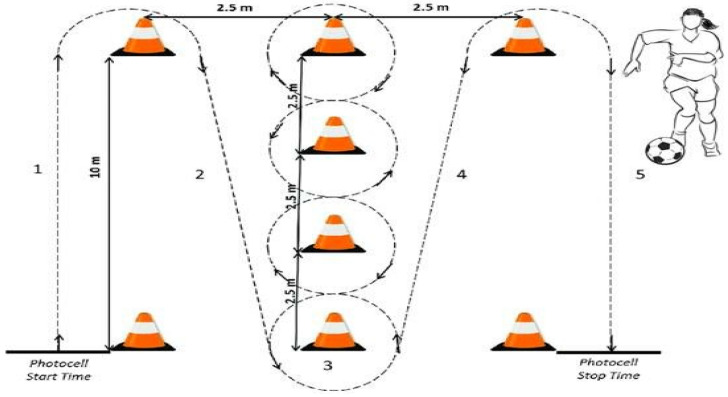
Illinois Agility Run Test [27]. 1. Starting Point: The course is set up in a flat area measuring 10 m in length and 5 m in width.The starting line marks the participant’s initial position. 2. Straight Run Area: The participant runs straight for 10 m from the starting point. 3. Slalom Zone: Four cones are placed between the 3rd and 7th meters to facilitate zigzag (slalom) running. The cones are spaced 3.3 m apart. 4. Return: After completing the slalom section, the participant runs straight back 10 m to the starting line. 5. Finish Line: The course is completed when the participant crosses the finish line, and the time is recorded.

The Illinois Agility Test (IAT) was employed to assess the players’ agility and ability to navigate rapid directional changes on the field. The test utilized a setup consisting of eight cones and two pairs of photocells (Autonics, BX5M—MFR—T, Seongnam, Republic of Korea), which were strategically positioned to evaluate the participants’ agility under controlled conditions. The cones were arranged in a rectangular formation, measuring 10 m in length and 5 m in width. The layout included two cones marking the starting and finishing points, while the remaining four cones served as markers for the turning points, which are critical to testing the participant’s ability to decelerate, change direction, and accelerate efficiently. The interior cones were aligned in a straight line, with a spacing of 3.3 m between each cone. This arrangement allowed for a series of rapid changes in direction as the participants weaved through the course, simulating movements typically performed during a match. The configuration of the cones was designed to challenge players’ speed, balance, and coordination, as they were required to accelerate and decelerate quickly while maintaining control and fluidity of motion. The participants initiated the test by placing one foot on the starting line in a standing position. As soon as the first set of photocells was crossed, the timing for the test began. Notably, the test was designed to eliminate any external variables related to reaction time by not using an audible start signal. This allowed each player to begin at their own pace, reducing the potential impact of reaction time on the test results. By giving the participants the freedom to start when they felt ready, the test more accurately reflected their natural movement patterns and decision-making skills in response to their environment, rather than focusing on reaction time alone. The Illinois Agility Test is widely regarded as a reliable and valid measure of agility in athletes, particularly in sports like soccer where rapid changes of direction are critical to performance. The test assesses both physical and cognitive components, as athletes must process visual information, make split-second decisions, and execute quick movements (Figure 2). By employing this test in the study, we were able to evaluate the players’ agility in a way that simulates the dynamic demands of a soccer match. [27].

#### 2.5.3. Cardiorespiratory Fitness—Yo-Yo Intermittent Recovery Test Level 1

**Figure 3:**
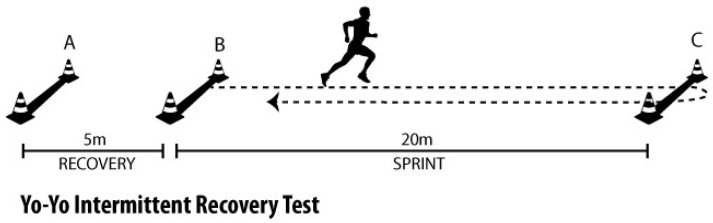
Yo-Yo Intermittent Recovery Test Level 1 [30]. B. Starting Point: The course is set up on a straight area measuring 20 m in length. The starting line marks the point where the run begins. B-C. Running Distance (20 Meters): The participant starts running straight for 20 m after the start signal. C-B. Turning Line: At the end of the 20 m, the participant reaches the turning line and then returns to the starting line. A. Recovery Area (5 Meters): After returning to the starting line, the participant walks 5 m into the recovery area. The participant rests for a set amount of time (determined by the test level) in the recovery area.

The Yo-Yo Intermittent Recovery Test Level 1 (Yo-Yo IRT1) was conducted following well-established protocols designed to assess an athlete’s intermittent endurance capacity, which is crucial for sports such as soccer, where players frequently alternate between high-intensity sprints and brief recovery periods. The test consists of 20 m shuttle runs, with the running speed incrementally increasing as the test progresses. The test protocol comprises two sets of 20 runs, each executed at progressively higher speeds, controlled by auditory cues provided through an iPad (Apple Inc., New York, NY, USA) linked via Bluetooth to a speaker (Xaomi Inc., MDZ, Beijing, China), placed next to the 20 m finish line. At the commencement of each run, participants begin at the starting line and sprint to a cone placed 20 m away, at which point they execute a 180-degree pivot before returning to the start. This shuttle-based format closely replicates the demands of team sports, where athletes are required to rapidly change direction and recover between bouts of intense running. The initial set of runs begins at speeds between 10 and 13 km/h, and participants are then required to maintain a running pace at speeds of 13.5–14 km/h during the second set. After completing every eighth run, the speed was increased by 0.5 km/h until participants could no longer maintain the required pace. A 10 min rest interval was provided between each phase of the test, allowing for partial recovery, mirroring the brief recovery periods found in sports like soccer. The participants’ endurance was assessed based on the total distance covered during the test, reflecting the athlete’s ability to perform at varying intensities over extended periods. If a participant was unable to complete the full distance required for any given stage, the test continued, and the distance achieved up until the point of failure was recorded for performance evaluation (Figure 3).

The Yo-Yo IRT1 is widely considered one of the most reliable and validated methods for measuring aerobic endurance and intermittent running capacity in athletes. Previous studies have demonstrated its high reproducibility, with an Intra-class Correlation Coefficient (ICC) of 0.98 and a Coefficient of Variation (CV) of 3.5%, both indicating excellent consistency and precision in the test outcomes. These reliability measures are crucial for ensuring the test’s accuracy in assessing athletes’ cardiovascular and muscular endurance capabilities under conditions that closely simulate the physical demands of soccer matches, where players are frequently required to perform short bursts of high-intensity sprints interspersed with brief recovery periods.

In this study, the maximal oxygen consumption (VO_2_ max) of participants was estimated using the following formula, derived from the total distance covered during the Yo-Yo IRT1: Yo-Yo IRT1: VO_2_ max (mL ∗ kg⁻¹ ∗ min⁻¹) = Yo-Yo 1 distance (m) ∗ 0.0084 + 36.4

This formula provides an estimation of VO_2_ Max, a key physiological parameter that reflects an athlete’s ability to take in, transport, and utilize oxygen during intense physical exertion.

VO_2_ max is widely regarded as the gold standard for assessing aerobic endurance and is a strong predictor of performance in endurance-based sports. Given the importance of aerobic capacity in soccer, this method of estimating VO_2_ max is particularly relevant for evaluating young professional players, who must meet high demands for endurance and recovery during matches. Overall, the Yo-Yo IRT1 offers a comprehensive and effective evaluation of intermittent endurance performance, providing valuable insight into an athlete’s cardiovascular fitness and recovery ability, which are critical components of performance in team sports like soccer. By simulating the specific movement patterns and intensities experienced during matches, the test serves as an excellent tool for assessing players’ aerobic fitness levels and tailoring training programs aimed at improving their performance in competitive environments [28,29,30].

### 2.6. Proprioceptive Training Program and Research Design

This study aimed to evaluate the effect of a 12-week proprioceptive training program on the technical soccer skills of young athletes. The program consisted of five training sessions per week, ensuring regular and consistent stimulus for the development of proprioception and related motor skills. A structured, 15 min standardized warm-up was conducted prior to each training session to prepare the participants both physically and mentally. The warm-up focused on general physical conditioning and dynamic stretching, enhancing flexibility and mobility, thus optimizing the participants’ readiness for the proprioceptive exercises that followed. Each proprioceptive training session lasted approximately 20 min and was designed to progressively challenge the athletes’ balance, coordination, and body control. To maintain consistency and minimize the potential influence of external variables, all sessions were scheduled to begin at 10:00 a.m. This time was selected to reduce potential discrepancies caused by diurnal fluctuations in physical performance. By maintaining this consistent starting time for each session, the researchers aimed to standardize the environmental and physiological conditions under which the athletes performed. The proprioceptive training program incorporated a variety of exercises specifically designed to enhance balance and coordination, key components for improving technical soccer skills. These exercises included agility ladder drills, circle drills, and exercises using BOSU balls, which are effective tools for enhancing dynamic balance and core stability. Additionally, participants engaged in training with TOGU balls and inflatable balance discs, which provide an unstable surface, forcing the body to adapt by strengthening proprioceptive pathways and improving postural control. Furthermore, mini trampoline exercises were included to improve reactive strength and enhance coordination, as the bouncing motion on a trampoline challenges the body’s ability to stabilize and control rapid movements. The diverse set of exercises was strategically selected to target a wide range of proprioceptive abilities, providing comprehensive development of balance, agility, and coordination. These abilities are foundational for technical soccer skills, such as dribbling, passing, and shooting, which require rapid adjustments to body position and movement patterns in response to both the player’s own actions and the evolving dynamics of the game. By systematically exposing the athletes to a series of progressively challenging proprioceptive exercises, the training program aimed to improve their technical performance on the soccer field, fostering enhanced control over their movements, quicker reactions, and more efficient execution of soccer skills. The structure and design of the training program were intended to not only enhance physical capabilities but also to contribute to the development of skills directly applicable to the demands of competitive soccer (Table 3). [19].

#### 2.6.1. Agility Ladder Exercises

In this study, the agility ladder used for proprioceptive training measured 5.55 m in length and 0.50 m in width, designed with unbreakable plastic rungs connected by durable straps to withstand intense use. The ladder was positioned on the ground in a manner that ensured the exercises could be performed with consistency and safety. Participants stood 50 cm behind the ladder in an upright position, preparing to execute a series of dynamic agility drills. The exercises were designed to target specific movement patterns and develop both lower body agility and coordination, crucial components for improving soccer-specific skills.

The series of exercises performed included the following:

Forward Sprint with One Foot per Square: Participants sprinted forward, placing one foot in each square of the ladder. This drill focused on developing quick, controlled steps and enhancing foot speed, which is essential for rapid directional changes in soccer.

Forward Sprint with Two Feet per Square: In this variation, athletes sprinted forward, placing both feet into each square of the ladder. This drill aimed to improve overall sprinting speed while maintaining control and precision during rapid forward movements.

Forward Straddle Hops: Participants hopped forward, straddling the ladder with each jump. This exercise worked on explosive lower body power, requiring the athletes to engage their leg muscles in an explosive, quick movement, mimicking the explosive sprints and directional changes in soccer.

Lateral Movements: In this drill, athletes stood beside the ladder and moved laterally in and out of the rungs. Lateral agility is crucial in soccer for defensive and offensive maneuvers, as players often need to quickly shift their weight and change direction horizontally on the field.

Single-Leg Linear Jumps: This drill required players to jump forward using only one leg at a time. It enhanced unilateral leg strength, stability, and control, which are essential for maintaining balance while executing dynamic actions like cutting and pivoting during a game.

Single-Leg Lateral Jumps: Similar to the linear jump, this drill involved jumping laterally on one foot. It focused on improving lateral balance, coordination, and strength in one leg, all of which are key for sidestepping opponents and making quick changes of direction.

Slalom Jumps: Athletes performed quick jumps in a slalom pattern, placing one foot in each square of the ladder. This exercise focused on improving the player’s agility and coordination in unpredictable, changeable directions, simulating rapid shifts in movement that occur during a soccer match.

Cross-Slalom Jumps: In this more advanced variation, players performed cross-slalom jumps across the ladder, enhancing their ability to make complex, rapid directional changes while maintaining balance and speed.

All exercises were performed at a high intensity, with the goal of improving speed, agility, and coordination in a soccer-specific context. After completing the ladder drills, a soccer ball was placed one meter beyond the end of the ladder, and participants were required to immediately transition into shooting drills. Three small goals, each measuring 1 m in length and 60 cm in height, were set up 10 m away from the ball. The goals were strategically positioned: two diagonally and one directly ahead of the ball, mimicking the spatial setup of real-game scenarios where players need to quickly adjust their aim and scoring technique after rapid movements. After finishing the ladder drills, players aimed to shoot and score in one of the three goals, which added a technical soccer skill component to the agility training, ensuring the training had a functional transfer to game-like situations. This combination of proprioceptive training with both agility drills and technical soccer skills aimed to improve the players’ overall performance, helping them develop enhanced movement efficiency, rapid decision-making, and precise ball control—all critical elements of soccer performance at the professional level.

#### 2.6.2. Circle Exercises

In this segment of the proprioceptive training, 24 circles, each with a diameter of 80 cm, were strategically arranged to form two distinct paths. The setup consisted of 12 circles per path, creating two parallel courses designed to challenge the players’ agility, coordination, and ability to maintain control under dynamic conditions. One path followed a straight line of circles, arranged in a single file. This arrangement was intended to challenge players’ ability to move efficiently in a linear fashion, requiring quick footwork and precise placement within each circle. The second path employed a two-one-two pattern, which required athletes to navigate in a series of alternating steps, mirroring more complex movement patterns frequently encountered in soccer matches, such as changes in direction or evasion of opponents. The exercises performed in the circle course mirrored those from the agility ladder drills, including the following:

Forward Movement: Players quickly stepped into each circle in rapid succession, either placing one or both feet in each circle, focusing on maintaining foot speed and body control.

Lateral Movement: Athletes moved laterally from one circle to the next, similar to the lateral movements in the ladder drills, challenging their ability to shift their weight and maintain balance while rapidly changing direction.

In-and-Out Steps: This drill required players to step in and out of each circle, mimicking quick directional shifts and enhancing coordination, which is crucial for maintaining control when moving between opponents on the field.

After completing the circle drills, players were instructed to rapidly dribble the ball for 10 m, transitioning smoothly from the circle course to the ball control task. The ball was placed at the end of the path, and athletes were required to demonstrate their ball-handling skills in a dynamic situation, simulating the need for quick transitions from movement drills to game-specific actions. Following the dribbling task, players were tasked with attempting to score a goal using their preferred foot in one of three small nets, positioned as in the previous section. This final step integrated technical soccer skills into the proprioceptive training program, ensuring that the training drills were not only improving agility and coordination but also directly transferable to real match situations, where quick decision-making, ball control, and precise shooting are essential. Overall, this training session aimed to enhance the players’ ability to execute rapid movements, maintain agility under pressure, and efficiently transition between different types of actions, all while improving their technical soccer skills in a game-like context.

#### 2.6.3. BOSU Exercises

The BOSU balance trainer (BOSU, Canton, OH, USA) was employed to enhance balance in the participants. Players attempted to stabilize themselves while standing on the inflatable portion of the BOSU, which measured approximately 25 cm in height, using one foot (either dominant or non-dominant). After receiving a signal from a teammate, players executed the prescribed exercises in succession.

Single-Leg Balance: Participants were instructed to maintain their balance on the BOSU for a duration of 30 s, alternating between their dominant and non-dominant legs with both arms placed on their hips.

Foot Return Drill: While balancing on the BOSU for 30 s, a teammate threw a ball from a distance of 5 m.

The player was required to return the ball using the inside (and/or top) of their foot without allowing the ball or their foot to touch the ground.

Header Return Drill: This exercise was similar to the previous one, but the player was required to return the ball using a header.

Juggling Exercise: This drill replicated the second exercise but required players to juggle the ball as many times as possible during the 30 s interval without allowing it to touch the ground. Participants could utilize various body parts to keep the ball airborne.

Goal Shooting Drill: This exercise was akin to the second one, but players had to shoot at one of three small goals, placed at a diagonal distance of 10 m from the ball and arranged vertically (each goal measuring 1 m in length and 60 cm in height). Each exercise lasted for 30 s, followed by a 30 s rest period. During the proprioceptive training sessions, players alternated their use of dominant and non-dominant legs. If a player lost balance or touched the ground, they were required to reset and maintain their position on the BOSU until the 30 s concluded. In addition, participants performed the same exercises on three alternative types of equipment: (1) a TOGU© Redondo ball, with a base diameter of 40 cm and a height of approximately 20 cm, (2) inflatable balance discs (with a base diameter of 33 cm and height of about 20 cm), and (3) mini-trampolines (30 cm in height and 1.4 m in diameter) [31].

### 2.7. Soccer Training Phases

To provide sufficient space for practice across the entire soccer field, six training phases were established. Each phase consisted of two stations: two stations for agility ladder exercises, two for hoop exercises, two for BOSU exercises, two for TOGU exercises, two for inflatable discs, and two for trampoline exercises.

To maximize practice time efficiency, players were divided into 12 pairs (1 pair per station). Each pair was required to practice for a duration of 3 min at each station. In stations where players were required to receive the ball from their teammates, they were instructed to switch roles every 30 s. All participants rotated through the same number of stations, and the sequence of intervention exercises was presented in a randomized order for all participant pairs [32].

#### 2.7.1. Evaluation of Soccer Technical Skills

Reliable and distinguishable measurements are generally recognized as essential for assessing changes in soccer performance over time [32,33,34,35]. The outcomes of a training intervention program provide coaches with valuable insights into the improvement of players’ performance [36].

In line with the objectives of this study, pre- and post-intervention evaluations were conducted specifically focusing on the targets of proprioceptive training, and assessing technical soccer skills through juggling, shooting, and agility tests.

Juggling Tests: A series of juggling tests was conducted to evaluate the coordination of soccer players. Each participant had three attempts to perform as many juggle touches as possible without letting the ball touch the ground. A maximum score of 100 contact points was established for both the freestyle juggling (body) and alternating foot juggling tests. Each attempt began with the ball being released from the hands to the feet. The procedures were similar to those outlined by [37]; however, there were some differences in performance testing and evaluation, which are described below.

Free Juggling (Body) Test: Participants were required to perform three juggling attempts, during which the ball could make contact with various body parts. The order of juggling was left to the participants’ discretion and comfort. The measurement unit was defined as 1 point for each ball contact. The highest score obtained from the three attempts was used for further analysis [37].

Alternating Foot Juggling Test: Participants were required to juggle the ball in a specific sequence, alternating between right and left foot (right-left-right or left-right-left). The ball was not allowed to touch the ground or any other body parts. The measurement unit was defined as 1 point for each successful juggling action. The highest score obtained from the three attempts was used for further analysis [37].

Juggling (Foot) Test: Participants performed three juggling attempts with their right foot and three with their left foot. The measurement unit was established as 1 point for each successful juggling action. The highest score obtained from the three attempts for both the right and left foot was used for further analysis [37].

Shooting Test: This test was a modified version of the shooting (dead ball) test by Rösch et al. and was utilized to evaluate shooting accuracy and coordination. Players were required to shoot the ball into a standard goal divided into six segments. The ball was positioned 16.5 m away from the center of the goal. Participants were instructed to aim for the upper left and/or upper right segments, where they could achieve the highest scores, and to complete five attempts. The total points from the five attempts were recorded for further analysis [32,33,34,35,36,37]. All intervention exercises and evaluation tests were conducted on natural grass, and players wore soccer cleats. Participants also performed a practice trial to familiarize themselves with the assessment tests.

#### 2.7.2. Methodological Considerations and Future Research Directions

One of the main limitations of this study lies in the small sample size, which raises concerns regarding the generalizability of the findings. However, a comprehensive review of the literature indicates that previous research on proprioceptive exercises for soccer players has primarily focused on various warm-up and stretching protocols aimed at enhancing performance factors such as anaerobic capacity, agility, dribbling, and shooting [21,22,23,24]. In contrast, studies specifically investigating the effects of proprioceptive training on the physical fitness and technical performance of young soccer players remain relatively limited. This study, therefore, seeks to address this gap by examining the impact of proprioceptive training on both the physical fitness and soccer-specific technical skills of young athletes.

Although the sample size in this study is relatively small, the findings offer valuable insights that can inform future research with larger sample sizes and more diverse populations. To improve the generalizability of the results, future studies should consider including participants from a broader range of skill levels (e.g., amateur and elite players), various age groups (such as young, middle-aged, and older athletes), and both male and female athletes. Additionally, the impact of cultural and geographical differences on the outcomes of proprioceptive training should be explored through cross-cultural studies. Conducting similar research in diverse cultural contexts will further assess the universal applicability of proprioceptive training interventions, enhancing the external validity of the findings.

### 2.8. Ethical Approval

The Ethical approval was obtained from the Health Sciences Ethics Committee of Çankırı Karatekin University, with the application submitted under meeting number 12 on 19 March 2024 (code 76fb8a54f90e4d6c). Participants provided informed consent, which included comprehensive details about the research, associated risks, potential benefits, confidentiality measures, and participant rights. The study strictly adhered to the ethical principles outlined in the Declaration of Helsinki, ensuring the protection of participants’ rights and well-being throughout the design, procedures, and confidentiality measures. All stages of this study complied with the Helsinki guidelines for human research and met the current ethical standards in Sport and Exercise Science.

### 2.9. Statistical Analysis

The data were analyzed using the SPSS 22 statistical software. Normality tests were conducted utilizing the Shapiro–Wilk test, and additional assessments included skewness-kurtosis values, histograms, box plots, and Q-Q plots. Given that the data adhered to a normal distribution, results are presented as mean ± standard deviation (± SD). Paired sample *t*-tests were employed for binary comparisons. To demonstrate the practical significance of differences between pre-test and post-test results, effect sizes and percentage changes were reported. Effect size was determined using Cohen’s d test, with classification based on Hopkins’ 2002 classification table [38,39]. Percentage changes (%Δ) between measurements were calculated using the formula presented in Figure 1 [15,40]. To assess the interaction effect (group x time), a two-way repeated measures ANOVA was utilized. The eta squared (η^2^) value was employed to express the effect size, categorized as small ≥ 0.01, medium ≥ 0.06, and large ≥ 0.14 [41]. The reliability level was set at 95%, and the significance level was interpreted at *p* = 0.05.

%Δ = (Pre Test − Post Test)/(Pre Test) × 100

This formula calculates the percentage change between pre-test and post-test values, providing insight into the magnitude of change resulting from the intervention.

## 3. Results

The findings obtained within the scope of the study are presented below. This section presents the analyses of the collected data. The findings are displayed in tabular form. The practical significance of the effects of proprioceptive training on physiological characteristics is illustrated in Table 4.

In Table 4, the findings regarding the practical significance of the effects of proprioceptive training on physiological characteristics are presented. According to the results, the effect sizes calculated using Cohen’s d for the effects of proprioceptive training on weight (kg) were found to be medium for the control group (d = 1.18, %Δ = 5.02) and very large for the experimental group (d = 2.04, %Δ = 7.76). For body mass index, the effect sizes were medium for both the control group (d = 1.02, %Δ = 5.00) and the experimental group (d = 1.06, %Δ = 7.59). In terms of body fat percentage, the effects were very large for both groups: control group (d = 2.05, %Δ = 9.77) and experimental group (d = 2.97, %Δ = 17.58). Finally, the effects on maximum oxygen consumption were medium for the control group (d = 0.63, %Δ = 1.86) and medium for the experimental group (d = 1.18, %Δ = 4.11).

Table 5 presents findings regarding the practical significance of the effects of proprioceptive training on technical skills and various performance parameters in football.

In Table 5, the findings regarding the practical significance of proprioceptive training effects on technical skills and certain performance parameters in football are presented. According to the results, based on the Cohen’s d coefficient calculated to determine the effect size of the differences, the effects of proprioceptive training on free juggling were insignificant for the control group (d = 0.13, %Δ = 0.33), but large for the experimental group (d = 1.69, %Δ = 4.61); the effects on alternating foot juggling were moderate for the control group (d = 1.00, %Δ = 12.79) and very large for the experimental group (d = 2.75, %Δ = 34.54); the effects on juggling with the right foot were moderate for the control group (d = 0.75, %Δ = 3.58) and large for the experimental group (d = 1.94, %Δ = 9.54); the effects on juggling with the left foot were large for both the control group (d = 1.28, %Δ = 5.90) and the experimental group (d = 1.38, %Δ = 6.38); the effects on shooting were large for the control group (d = 1.22, %Δ = 10.11) and very large for the experimental group (d = 2.81, %Δ = 32.36); and the effects on the Illinois agility test were small for the control group (d = 0.27, %Δ = 1.48) and moderate for the experimental group (d = 1.06, %Δ = 3.25)

The findings comparing the effects of proprioceptive training on physiological characteristics between groups over time are presented in Table 6.

In Table 6, the findings of the repeated measures two-way ANOVA test comparing the effects of proprioceptive training on the physiological characteristics of football players over time between the control and experimental groups are presented. According to the results, a statistically significant difference was found between the groups over time only in body fat percentage (BF%) (*p* < 0.05; η² = 0.006).

The findings comparing the effects of proprioceptive training on technical skills and certain performance parameters in football between groups over time are presented in Table 7.

In Table 7, the findings of the repeated measures two-way ANOVA test comparing the effects of proprioceptive training on the technical skills and certain performance parameters of football players over time between the control and experimental groups are presented. According to the results, statistically significant differences were found between the groups over time in free juggling (*p* < 0.05; η^2^ = 0.302), alternating foot juggling (*p* < 0.05; η^2^ = 0.271), juggling with the right foot (*p* < 0.05; η^2^ = 0.250), and shooting performance (*p* < 0.05; η^2^ = 0.513).

## 4. Discussion

The aim of this study was to investigate the effects of a 12-week proprioceptive training program on the physical–physiological fitness and soccer-specific technical skills of young soccer players, while also considering its potential impact on injury prevention and rehabilitation. The experimental group, which underwent proprioceptive training, showed significant improvements in body fat percentage and certain soccer-specific technical skills, such as free juggling, foot-switch juggling, and shooting. These findings are consistent with previous research indicating that proprioceptive training enhances athletes’ physical fitness, technical performance, and injury resistance [42,43].

However, improvements in agility performance and other physical parameters, such as maxVO_2_ capacity, were either minimal or absent. This could be due to the design of the training protocol and the limited focus on specific physical attributes. The results suggest that proprioceptive training had no significant impact on agility and cardiovascular fitness. This may be due to insufficient training duration and intensity for these parameters, or alternatively, the need for different test methods targeting these aspects. The literature recommends different training approaches that may be more effective for enhancing agility and cardiovascular fitness [1,2,6,7].

### 4.1. Participant Profile and Generalizability

This study was conducted with a limited sample of professional athletes from a specific age group, which restricts the generalizability of the findings. The participant profile limits the applicability of the results. Future research should involve athletes from various age groups, skill levels, and genders to expand the scope and increase the reliability of the findings. Moreover, when the study group includes a broader and more heterogeneous population, the reliability of the results in relation to general athletic populations can be further assessed.

### 4.2. Proprioceptive Training and Physical-Fitness Parameters

The positive effects of proprioceptive training on reducing body fat percentage are consistent with findings suggesting that such training can improve muscle activation, body composition, and neuromuscular control [43,44]. These benefits are particularly important for young athletes, as reducing body fat and increasing muscle mass may help reduce the risk of overuse injuries commonly observed among soccer players. Additionally, proprioceptive training may assist in injury rehabilitation by strengthening weak or injured muscle groups, and restoring proper alignment and posture [45]. Increased neuromuscular control plays a critical role in reducing injury risk, especially in sports like soccer that involve sudden movements and joint stress.

While significant improvements in body fat percentage were observed, no meaningful changes were seen in maxVO_2_ capacity. This is consistent with previous findings indicating that proprioceptive training primarily improves balance, coordination, and stability, but does not directly affect aerobic endurance [46]. The literature emphasizes that endurance training is more effective in enhancing aerobic capacity [6]. Since soccer players require both aerobic endurance and neuromuscular control, combining proprioceptive training with endurance interventions could be more effective in improving overall physical fitness and preventing cardiovascular strain during high-intensity matches.

### 4.3. Proprioceptive Training and Soccer-Specific Technical Skills

The improvements observed in soccer-specific technical skills in this study are particularly noteworthy. Proprioceptive training has been shown to enhance motor coordination and technical proficiency, leading to more efficient movement patterns and a reduction in injury risk [47]. For instance, improvements in free juggling, foot-switch juggling, and shooting suggest that players gained better body awareness, spatial orientation, and postural stability, all of which are crucial factors in reducing injury risk. Proprioceptive recovery also enables athletes to recover more effectively from unbalanced positions, thereby minimizing the risk of muscle strains or ligament injuries during sudden movements.

Interestingly, significant improvements were observed in skills performed with the right foot, while no changes were noted for the left foot. This highlights the importance of focusing on both sides of the body during training. The lack of improvement in the non-dominant side may indicate neuromuscular asymmetry, which could increase the risk of overuse injuries on the dominant side, particularly when athletes frequently use one foot for movements such as ball control and shooting. This observation suggests that proprioceptive training should be tailored to promote balanced development of both limbs, preventing overloading of one side, which could reduce the incidence of overuse injuries, such as tendinitis or stress fractures [48].

### 4.4. Agility Performance and Proprioceptive Training

One of the most debated results in this study concerns the effects of proprioceptive training on agility performance. Agility is crucial for soccer players, as it involves rapid changes of direction. However, no significant improvement in agility was observed in this study, which is inconsistent with some previous research [49,50]. Agility requires not only neuromuscular control but also strength, speed, acceleration, and deceleration abilities. While proprioceptive training improves balance and coordination, it may not directly target the neural adaptations required for these agility components, such as fast-twitch muscle fiber activation or explosive power. Furthermore, agility involves sport-specific exercises that require rapid and multidirectional movements, which may not have been sufficiently incorporated into the current proprioceptive training program [51,52,53].

### 4.5. Biomechanical and Neurophysiological Principles

The improvements in body fat percentage and soccer-specific technical skills can be attributed to enhanced neuromuscular control and muscle activation. Proprioceptive training improves the function of proprioceptors in muscles and joints, contributing to better body awareness, balance, and stability [54,55,56]. These improvements enable athletes to perform more efficiently while reducing compensatory movements that may lead to injury. From a biomechanical perspective, the training encourages better joint position sense and dynamic stability, which is crucial for executing complex movements like shooting and juggling under pressure [57,58]. Additionally, enhanced neuromuscular control increases joint stability during rapid movements, helping to reduce the risk of muscle strains or ligament injuries. However, the lack of significant improvement in agility and cardiovascular fitness can be explained by neurophysiological principles. Agility performance requires more than proprioceptive feedback; it also demands the ability to accelerate and decelerate rapidly, as well as explosive power. While proprioceptive training improves balance and stability, it may not directly target the neural adaptations required for agility, such as fast muscle fiber activation. Furthermore, agility involves sport-specific exercises, and the current proprioceptive training protocol may not have sufficiently included exercises that target this skill [59,60,61].

## 5. Conclusions

In conclusion, while proprioceptive training offers significant benefits in enhancing specific technical skills and improving body composition, its effectiveness in boosting agility and cardiovascular endurance appears limited. The study’s findings underscore that while proprioceptive training can improve aspects of neuromuscular control, such as muscle activation and joint stability, it may not be sufficient on its own to address all facets of athletic performance. Particularly, agility and cardiovascular endurance, which require additional components like explosive strength, speed, and aerobic capacity, were not significantly enhanced through proprioceptive exercises alone.

Future research should explore the combination of proprioceptive training with sport-specific drills and endurance exercises to create a more holistic training approach that targets all dimensions of athletic performance. By integrating sport-specific exercises, the benefits of proprioceptive training could be enhanced, particularly for agility, which demands rapid direction changes and dynamic movement patterns. Additionally, the inclusion of endurance training would be crucial to improving cardiovascular fitness, especially for sports like soccer that require sustained effort over long periods.

### 5.1. Applicability and Future Research

This study was conducted with 28 young male professional soccer players from Türkiye’s Professional Third League, aged approximately 20 years. The homogeneity of the sample limits the generalizability of the results, particularly as the findings may not apply to athletes from different age groups, genders, or sports backgrounds. Future studies should aim to include a more diverse group of participants, considering varying skill levels (from amateur to professional athletes), as well as different age groups, including young, middle-aged, and older athletes, to assess the effects of proprioceptive training across a broader spectrum of the athletic population. Furthermore, cultural differences may play a role in athletic performance, and thus, cross-cultural studies are recommended. By exploring how proprioceptive training protocols perform in different cultural contexts, such as in Western Europe or Asia, the research could offer insights into the universality and potential adaptations of these interventions. This would not only expand the applicability of the findings but also provide a deeper understanding of how such training affects athletes from various cultural and performance backgrounds.

Finally, future research should expand beyond professional soccer players to include a wider range of athletes from various sports disciplines. This will facilitate comparisons of how athletes from different sports and competitive levels respond to proprioceptive training, helping to draw more generalized conclusions regarding its effectiveness and potential for enhancing overall athletic performance.

### 5.2. Recommendations

#### 5.2.1. Integration with Other Training Methods

Proprioceptive training should be integrated into a broader athletic development program that includes components like endurance, strength, and agility. A comprehensive approach will ensure that all critical aspects of athletic performance are addressed, ultimately contributing to better performance outcomes and reduced injury risk. For instance, while proprioceptive exercises improve neuromuscular control and stability, they should be complemented with exercises targeting aerobic capacity and explosive power to foster overall athleticism and agility.

#### 5.2.2. Customized Rehabilitation Protocols

Rehabilitation protocols should be tailored to the specific needs of athletes, taking into account their baseline characteristics, injury history, and rehabilitation goals. For example, athletes recovering from lower limb injuries might benefit more from proprioceptive exercises focused on improving balance and joint stability. Customization of these protocols will help optimize rehabilitation outcomes and reduce the risk of re-injury.

#### 5.2.3. Long-Term Training and Rehabilitation

Future studies should focus on the long-term effects of proprioceptive training, particularly in injury rehabilitation. While this study demonstrates the short-term benefits of proprioceptive training, understanding its full potential in restoring movement function and preventing re-injury requires a longitudinal approach. Long-term research could provide more robust evidence on the lasting impacts of proprioceptive exercises on injury prevention and recovery, especially in sports that involve frequent high-impact movements like soccer.

#### 5.2.4. Agility-Specific Rehabilitation

To improve agility, proprioceptive training should be combined with sport-specific agility drills. Agility involves more than just balance and stability; it requires rapid changes of direction, acceleration, and deceleration. Proprioceptive exercises that improve balance and joint stability should be integrated with drills that challenge these specific aspects of agility, helping athletes respond faster and more effectively in dynamic game situations. These combined efforts would likely result in improved agility performance and overall athletic functionality.

In conclusion, integrating proprioceptive training with other training modalities and tailoring it to individual needs can significantly enhance its effectiveness. Future research should continue to explore the synergistic effects of combined training approaches, incorporating a diverse range of athletes and sports to broaden our understanding of proprioceptive training’s impact on athletic performance and injury prevention.

## Figures and Tables

**Table 1:** Baseline Characteristics of the Study Participants.

Characteristic	Overall (n = 28)	Control Group (n = 14)	Experimental Group (n = 14)
Age (years)	20.46 ± 1.60	20.81 ± 1.66	20.07 ± 1.49
Height (cm)	182.89 ± 5.01	183.29 ± 4.38	182.50 ± 5.72
Body Weight (kg)	73.34 ± 3.10	73.08 ± 3.85	73.60 ± 2.24
Soccer Training Experience (years)	Minimum 5 years	-	-
Medical History	No cardiopulmonary conditions; no medication use	Same as Overall	Same as Overall
Additional Program	None	Standard training	Proprioceptive training
Medical Examination	Cleared for professional soccer	Same as Overall	Same as Overall

**Table 2:** Experimental Test Setup.

Monday: All tests’ practical demonstration
Tuesday
10:00—Body composition measurements, İlionis agility test
15:00—Yo yo IR1 maxVO_2_ test
Thursday
09:00—Technical soccer skill tests
Proprioceptive Training Programs/12 Weeks (Total)
Study Conclusion and Analysis Evaluation

**Table 3:** Detailed design of the Proprioception training programs.

1. Week	2. Week	3. Week	4. Week	5. Week
Pre Test and Evaluation	5 min. Jogging+ warm-up 10 min. stretching + Proprioception training +5 min. Jogging+ cool down	5 min. Jogging+ warm-up 10 min. stretching + Proprioception training +5 min. Jogging+ cool down	5 min. Jogging+ warm-up 10 min. stretching + Proprioception training +5 min. Jogging+ cool down	5 min. Jogging+ warm-up 10 min. stretching + Proprioception training +5 min. Jogging+ cool down
**6. week**	**7. week**	**8. week**	**9. week**	**10. week**
5 min. Jogging+ warm-up 10 min. Stretching+ Proprioception training+ 5 min. jogging+ cool down	5 min. Jogging+ warm-up 10 min. Stretching+ Proprioception training+ 5 min. jogging+ cool down	10 min. Jogging+ warm-up 10 min. Stretching+ Proprioception training+ 5 min. jogging+ cool down	10 min. Jogging+ warm-up 10 min. Stretching+ Proprioception training+ 5 min. jogging+ cool down	10 min. Jogging+ warm-up 10 min. Stretching+ Proprioception training+ 5 min. jogging+ cool down
**11. week**	**12. week**	**13. week**	**14. week**	
10 min. Jogging+ warm-up 10 min. Stretching+ Proprioception training+ 5 min. jogging+ cool down	10 min. Jogging+ warm-up 10 min. Stretching+ Proprioception training+ 5 min. jogging+ cool down	10 min. Jogging+ warm-up 10 min. Stretching+ Proprioception training+ 5 min. jogging+ cool down	Post Test and Evaluation	

**Table 4:** Practical Significance of the Effects of Proprioception Training on Physiological Characteristics.

Variables	Group	Pre Test $\bar{X}$ ± SS	Post Test $\bar{X}$ ± SS	%Δ	Cohen d
BW (kg)	CG	73.08 ± 3.85	69.41 ± 2.15	5.02	1.18
	EG	73.60 ± 2.24	67.89 ± 3.27	7.76	2.04
BMİ (kg/m^2^)	CG	21.77 ± 1.22	20.68 ± 0.89	5.00	1.02
	EG	22.14 ± 1.20	20.46 ± 1.90	7.59	1.06
BFP (%)	CG	20.48 ± 1.15	18.48 ± 0.76	9.77	2.05
	EG	21.61 ± 1.68	17.81 ± 0.68	17.58	2.97
maxVO_2_ (mL/kg/dk)	CG	51.72 ± 1.26	52.68 ± 1.74	1.86	0.63
	EG	52.09 ± 2.37	54.23 ± 1.01	4.11	1.18

Effect Size (Cohen d): Insignificant < 0.2; Small = 0.2–0.59; Moderate = 0.60–1.19; Large = 1.20–1.99; Very Large = 2.0–3.99; Close to Perfect > 4.0, BW: Body weight, BMİ: Body Mass İndex, BFP: Body Fat Percentage, CG: control group, EG: experimental group.

**Table 5:** Practical Significance of Proprioceptive Training Effects on Technical Skills and Performance Parameters in Soccer.

Variables	Group	Pre Test $\bar{X}$ ± SS	Post Test $\bar{X}$ ± SS	%Δ	Cohen d
Free juggling (points)	CG	42.50 ± 0.85	42.64 ± 1.22	0.33	0.13
	EG	43.43 ± 1.50	45.43 ± 0.76	4.61	1.69
Alternating foot juggling (points)	CG	25.65 ± 1.22	28.93 ± 4.46	12.79	1.00
	EG	24.00 ± 1.41	32.29 ± 4.03	34.54	2.75
Juggling with right foot (points)	CG	17.86 ± 0.66	18.50 ± 1.02	3.58	0.75
	EG	17.93 ± 0.92	19.64 ± 0.84	9.54	1.94
Juggling with left foot (points)	CG	18.14 ± 1.10	19.21 ± 0.43	5.90	1.28
	EG	17.86 ± 0.66	19.00 ± 0.96	6.38	1.38
Shooting (points)	CG	5.64 ± 0.50	6.21 ± 0.43	10.11	1.22
	EG	5.50 ± 0.52	7.28 ± 0.73	32.36	2.81
İllinois Agility (secs.)	CG	18.19 ± 1.01	18.46 ± 0.99	1.48	0.27
	EG	17.85 ± 0.57	18.43 ± 0.52	3.25	1.06

Effect Size (Cohen d): Insignificant < 0.2; Small = 0.2–0.59; Moderate = 0.60–1.19; Large = 1.20–1.99; Very Large = 2.0–3.99; Close to Perfect > 4.0, CG: control group, EG: experimental group.

**Table 6:** Comparison of differences in physiological characteristics between groups over time.

Variables	Group	Pre Test $\bar{X}$ ± SS	Post Test $\bar{X}$ ± SS	Group × Time Interaction		
				F	*p*	η^2^
BW (kg)	CG	73.08 ± 3.85	69.41 ± 2.15	1.998	0.169	0.071
	EG	73.60 ± 2.24	67.89 ± 3.27			
BMİ (kg/m^2^)	CG	21.77 ± 1.22	20.68 ± 0.89	1.950	0.174	0.070
	EG	22.14 ± 1.20	20.46 ± 1.90			
BFP (%)	CG	20.48 ± 1.15	18.48 ± 0.76	9.004	0.006 *	0.257
	EG	21.61 ± 1.68	17.81 ± 0.68			
maxVO_2_ (mL/kg/dk)	CG	51.72 ± 1.26	52.68 ± 1.74	1.892	0.181	0.068
	EG	52.09 ± 2.37	54.23 ± 1.01			

* *p* < 0.05; η^2^: small > 0.01, medium ≥ 0.06, large ≥ 0.14, CG: control group, EG: experimental group.

**Table 7:** Comparison of differences in technical skills and certain performance parameters in football between groups over time.

Variables	Group	Pre Test $\bar{X}$ ± SS	Post Test $\bar{X}$ ± SS	Group × Time Interaction		
				F	*p*	η^2^
Free juggling (points)	CG	42.50 ± 0.85	42.64 ± 1.22	11.267	0.002 *	0.302
	EG	43.43 ± 1.50	45.43 ± 0.76			
Alternating foot juggling (points)	CG	25.65 ± 1.22	28.93 ± 4.46	9.687	0.004 *	0.271
	EG	24.00 ± 1.41	32.29 ±4.03			
Juggling with right foot (points)	CG	17.86 ± 0.66	18.50 ± 1.02	8.680	0.007 *	0.250
	EG	17.93 ± 0.92	19.64 ± 0.84			
Juggling with left foot (points)	CG	18.14 ± 1.10	19.21 ± 0.43	0.032	0.859	0.001
	EG	17.86 ± 0.66	19.00 ± 0.96			
Shooting (points)	CG	5.64 ± 0.50	6.21 ± 0.43	27.423	0.001 *	0.513
	EG	5.50 ± 0.52	7.28 ± 0.73			
İllinois Agility (secs.)	CG	18.19 ± 1.01	18.46 ± 0.99	0.394	0.536	0.015
	EG	17.85 ± 0.57	18.43 ± 0.52			

* *p* < 0.05; η^2^: small > 0.01, medium ≥ 0.06, large ≥ 0.14, CG: control group, EG: experimental group.

## Data Availability

The raw data supporting the conclusions of this article will be made available by the authors on request.
